# Unsaturated phosphatidylcholines lining on the surface of cartilage and its possible physiological roles

**DOI:** 10.1186/1749-799X-2-14

**Published:** 2007-08-23

**Authors:** Yi Chen, Ross W Crawford, Adekunle Oloyede

**Affiliations:** 1 Orthopedic Research Unit, Level 5, Clinical Science Building, Prince Charles Hospital, Rode Road, Chermside, Q 4032, Australia; 2 School of Engineering Systems, Queensland University of Technology, Gardens Point Campus, P.O. Box 2434, 2 George Street, Brisbane Q 4001, Australia

## Abstract

**Background:**

Evidence has strongly indicated that surface-active phospholipid (SAPL), or surfactant, lines the surface of cartilage and serves as a lubricating agent. Previous clinical study showed that a saturated phosphatidylcholine (SPC), dipalmitoyl-phosphatidylcholine (DPPC), was effective in the treatment of osteoarthritis, however recent studies suggested that the dominant SAPL species at some sites outside the lung are not SPC, rather, are unsaturated phosphatidylcholine (USPC). Some of these USPC have been proven to be good boundary lubricants by our previous study, implicating their possible important physiological roles in joint if their existence can be confirmed. So far, no study has been conducted to identify the whole molecule species of different phosphatidylcholine (PC) classes on the surface of cartilage. In this study we identified the dominant PC molecule species on the surface of cartilage. We also confirmed that some of these PC species possess a property of semipermeability.

**Methods:**

HPLC was used to analyse the PC profile of bovine cartilage samples and comparisons of DPPC and USPC were carried out through semipermeability tests.

**Results:**

It was confirmed that USPC are the dominant SAPL species on the surface of cartilage. In particular, they are Dilinoleoyl-phosphatidylcholine (DLPC), Palmitoyl-linoleoyl-phosphatidylcholine, (PLPC), Palmitoyl-oleoyl-phosphatidylcholine (POPC) and Stearoyl-linoleoyl-phosphatidylcholine (SLPC). The relative content of DPPC (a SPC) was only 8%. Two USPC, PLPC and POPC, were capable of generating osmotic pressure that is equivalent to that by DPPC.

**Conclusion:**

The results from the current study confirm vigorously that USPC is the endogenous species inside the joint as against DPPC thereby confirming once again that USPC, and not SPC, characterizes the PC species distribution at non-lung sites of the body. USPC not only has better anti-friction and lubrication properties than DPPC, they also possess a level of semipermeability that is equivalent to DPPC. We therefore hypothesize that USPC can constitute a possible addition or alternative to the current commercially available viscosupplementation products for the prevention and treatment of osteoarthritis in the future.

## Background

Osteoarthritis is a disease that can compromise the functionality of the primary load-processing tissue of joints, namely articular cartilage. The cartilage tissue is covered by a thin layer of surface-active phospholipid (SAPL) of microscopic thickness that is believed to contribute to lubrication [1] and load processing [2]. Surface-active phospholipid is known more commonly as "surfactant" in the lung, where it is produced by alveolar Type II cells in the form of lamellar bodies, which are secreted onto the alveolar surface [3].

Studies indicate that SAPL is also synthesized and secreted in other parts of the body such as in the peritoneal cavity and joints [4,5], where its adsorption on the surfaces of tissues at these sites has been demonstrated using techniques such as electron microscopy, epifluorescence microscopy and autoradiography [6-8]. SAPL has also been found in many other non-lung sites of the body including the stomach and eustachian tube [9-11]. SAPL retains highly desirable physical and physiological properties, including: reduction of surface tension [1,12,13], physical barrier formation [11] and semipermeability [14].

ALEC™ is the only commercially available exogenous SAPL product that was developed initially for its clinical application in treating Respiratory Distress Syndrome (RDS) [15]. The main component of ALEC™ is a saturated phosphatidylcholine (SPC) called dipalmitoyl-phosphatidylcholine (DPPC) that is the main component of lung surfactant [3]. However, for a long time people had assumed that the main component of SAPL at the non-lung sites was also DPPC [1,8,9,11,13,15]. In these studies SAPL were always digested into inorganic phosphorus. By assuming all the phosphorus was coming from the DPPC molecules in the SAPL sample, the DPPC content was calculated by converting the amount of phosphorus into that of DPPC simply through the differences in their molecular weights. Subsequently several studies, including animal studies [16,17] and a clinical trial [18], have also been carried out to look at the efficacies of DPPC-based SAPL products in their treatment of different medical disorders at some non-lung sites. In particular, some promising results were noticed in the clinical trial in which a DPPC-based product was used to treat some osteoarthritis patients [18].

Two previous studies have shown that the dominant SAPL species at non-lung sites, such as the stomach and eustachian tube, are not SPC, but unsaturated phosphatidylcholine (USPC) [19,20]. Since then studies of USPC have been in two areas. The first is finding out real SAPL profiles at different non-lung sites such as the peritoneal cavity and joints. The second is comparing and examining whether USPC have the same or similar physical and physiological properties compared to DPPC. The information obtained from these two areas will be essential if we were to exploit the possibility of using appropriate SAPL for applications to alleviate medical disorders at non-lung sites. So far, it has been confirmed that the dominating SAPL species inside the peritoneal cavity are USPC and two dominating USPC species, palmitoyl-linoleoyl-phosphatidylcholine (PLPC) and palmitoyl-oleoyl-phosphatidylcholine (POPC), both of which possess anti-stick and lubrication properties similar to, if not better than those of DPPC [21]. Furthermore, the profile of SAPL species inside the pleural cavity has been found to be also dominated by USPC [22,23]. So far, there is no study identifying different species of SAPL bound to the cartilage surfaces at the molecular level, though Sarma et al. [24] tried to identify some PC species in an indirect way by analysing fatty acid chains attached to phosphatidylcholine backbones. However, the results from this study strongly suggested that USPC could be the dominating species inside the joint.

The semipermeability system inside the joint is important for fluid transport. As we already know, the PC lining on the surface of cartilage could serve not only as an effective lubricant but also as part of a whole semipermeable system to facilitate fluid transport at this site. It could be reasonably argued that when the PC lining on the cartilage surface becomes deficient the whole semipermeable system could be impaired, resulting in the abnormal accumulation of fluid inside the joint, causing joint effusion.

In this study we investigated the SAPL profile in the joint by analysing individual SAPL species as whole molecules. We also compared the semipermeability imparted by DPPC-based membranes to those made from particular USPC species. The outcomes from this study will further enhance our knowledge of SAPL profiles at non-lung sites. It will also aid our understanding of whether or not USPC species play any role in contributing to the physiological functions of the joint, leading to potential insight into the relationship between SAPL deficiency and articular cartilage function and degeneration.

## Methods

### Materials

Dipalmitoyl-phosphatidylcholine (DPPC), Dilinoleoyl-phosphatidylcholine (DLPC), Palmitoyl-linoleoylphosphatidylcholine, (PLPC), Palmitoyl-oleoyl-phosphatidylcholine (POPC), Dioleoyl-phosphatidylcholine (DOPC) and Stearoyl-linoleoylphosphatidylcholine (SLPC), Brij 35 (30% w/v), 1,6-diphenyl-1, 3, 5-hexatriene (DPH), and choline chloride were all analytic grade (AR) grade and were purchased from Sigma-Aldrich (Castle Hill, NSW, Australia). Methanol, acetonitrile and chloroform were HPLC grade purchased from EM Science (Merck, KGaA, Darmstadt, Germany).

### Preparation of bovine cartilage SAPL

Bovine cartilage phosphatidylcholines (PC) were extracted from the surface of ten articular cartilage specimens which were taken from the patellar grooves of 3–4 year old bovine animals harvested from the local abattoir on the experimental day. A standard lipid extraction procedure [25] was followed. The lipid solvent was chloroform: methanol (2:1), known as Folch reagent. During the collection procedure, soft facial tissues soaked with Folch solvent were used to wipe SAPL off from the articular surface. The contact time between the solvent and articular surface at each of these selected areas was all under 10 seconds as our pretest showed that this time frame did not cause any histological changes or damage to cartilage tissues. All used facial tissues were pooled together and soaked in Folch reagent. The chloroform phase containing both PC and non-PC species were obtained. The PC and non-PC species were then separated from each other by using a 100 mg Bondelut^R ^NH_2 _disposable cartridge column (Varian, Mulgrave, Vic., Australia), a standard method developed in a published study [26]. In brief, during this purification procedure, chloroform solution containing all the SAPL was allowed to pass through this cartridge. Since the particles inside the cartridge have a much stronger binding affinity to PC species than to non-PC species, only the PC component can be retained inside the cartridge and the non-PC component was eluted out of the cartridge. The cartridge was then washed with chloroform in order to eliminate any leftover non-PC component inside the cartridge. PC components were then eluted off by using chloroform/methanol (3:2, v/v). This chloroform/methanol solution containing PC species was used for subsequent HPLC assays.

### HPLC analysis

A 1100-series HPLC system (Agilent Technologies, Forest Hill, Vic., Australia) was used in combination with a RF-10AXL fluorescent detector (Shimadzu, Kyoto, Japan). Separations were screened on Phenosphere-NEXT C18 column (250 × 2 mm i.d., 5 μm particles) from Phenomenex Pty Ltd (Pennant Hills, NSW, Australia). The chromatographic conditions were based on those used in a published study [27]. The mobile phase was methanol (92.5% v/v) and water (7.5% v/v) with or without 40 mM choline chloride. The flow rate was 0.6 mL/min. The eluent was monitored by a fluorescent detector at 340/460 nm (excitation/emission) after post-column derivatization of mixed micelles with DPH using a 100 cm reaction coil at 50°C. The injection volume was 10 μl. The standard curves for all five PC species were all linear over the ranges of 5–25 mg/L and their correlation coefficients (r) were > 0.98. No internal standard was used. The inter-assay and intra-assay coefficients of variation were all < 10%. The recovery rates of all five PC species were > 80%. The detection limit for all five PC species was 50 ng. The relative percentages of each of the PC species were calculated after dividing their individual amount by the total PC amount.

### Measurement of semipermeability

Based on experience obtained from our previous study [14], 54 μl of individual synthetic PC (PLPC, POPC and DPPC) chloroform solution at a concentration of 21.85 mg/ml was deposited on each side of a disc of white nylon filter paper with a pore diameter of 0.2 μm (Millipore Corporation, Bedford, MA, U.S.A.). This was the carrier used to produce the PC semipermeable membrane. The solvent was removed by evaporation and the weight deposited per unit area was recorded as the effective "thickness". The achieved effective membrane thickness was 2.36 mg which was proven to be sufficient to cover the exposed area of 0.95 cm^2^.

**Figure 1:**
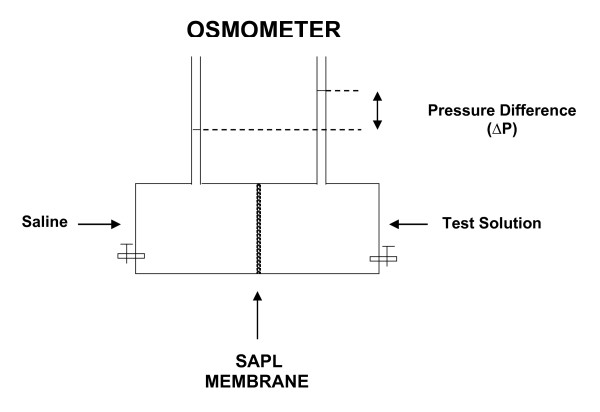
The structure of the 'osmometer' is illustrated, which consists of an Ussing chamber with two compartments holding test solution and saline separately, two vertical tubes connected to each of two compartments and used as osmotic pressure indicators, a SAPL membrane, and test/saline solutions. The "membrane" is clamped between the two compartments of an Ussing chamber. The test solution in the right compartment is "dialyzed" against saline in the left compartment.

Osmotic pressure was generated by clamping a PC membrane prepared as mentioned above between the two compartments of an Ussing chamber (Jim's Instrument Manufacturing, Iowa City, IA, U.S.A.). The left compartment was always filled with saline (sodium concentration of 0.15 M) and the right with hypertonic glucose solution (0.139 M) that was used in our previous similar study [14]. The total capacity of each compartment was approximately 0.7 ml, and the contact area between the two compartments was 0.44 cm^2^. 2.36 mg of DPPC or PLPC or POPC were used. Two vertical tubes with inner diameters of 1.2 mm were connected to the side of each compartment to measure osmotic pressure head. Figure 1 illustrates the device.

Osmotic pressure was measured as the difference in hydrostatic pressure of the compartments needed to stop further water transmission across the membrane. At the beginning of each experiment, the fluid heights indicating the pressure in both compartments, i.e. either sides of the membrane were set to the same level. The whole device was maintained at 37°C in a water bath, and the fluid heights indicating osmotic pressure difference (ΔP) were measured and recorded until no further movement of fluid was seen. At the end of each experiment, the final pressure difference, ΔP, was recorded as the difference in the heights between the two fluid columns. The mean and standard error of the mean (SEM) were calculated for each group of data points and the one-way ANOVA test was used for statistical analysis.

### Experimental procedure for osmosis testing

The experiment was divided into three sections:

Section I (n = 8): Measurement of osmotic pressure produced by dialyzing saline against hypertonic glucose solution (0.139 M) using a DPPC "membrane" of "thickness" 2.36 mg. Section II (n = 8): Measurement of osmotic pressure produced by dialyzing saline against hypertonic glucose solution (0.139 M) using a PLPC "membrane" of "thickness" 2.36 mg. Section III (n = 8): Measurement of osmotic pressure produced by dialyzing saline against hypertonic glucose solution (0.139 M) using a POPC "membrane" of "thickness" 2.36 mg.

## Results

Four USPC species and DPPC were identified from bovine cartilage samples assayed by our HPLC analysis. The total amount of PC was then worked out by adding the amounts of individual PC species together. In our study the total amount of PC species was < 20 μg. The relative percentages of each of the PC species were calculated after dividing their individual amount by the total PC amount. The individual relative percentages of these four USPC species were 23% for DLPC, 30% for PLPC, 17.5% for POPC and 16.0% for SLPC. The content of DPPC was found only to be 8%.

**Figure 2:**
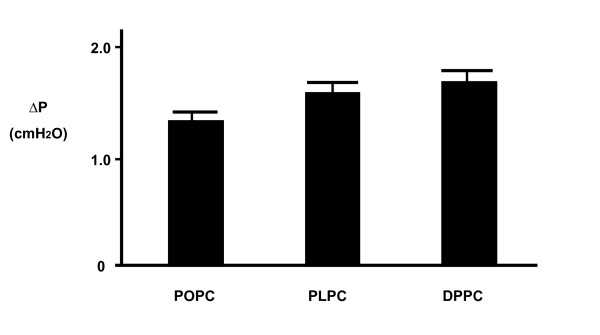
In this figure, the three bars represent the mean osmotic pressure difference (ΔP) generated by three SAPL "membranes", two of which were made with synthetic USPC ie POPC and PLPC, and one with synthetic SPC ie DPPC. There was no significant difference of ΔP between PLPC and DPPC 'membranes' was found (p < 0.05) though a significant difference was noticed between POPC and DPPC 'membranes' (p > 0.05).

In each of the eight runs using synthetic DPPC, PLPC and POPC membranes, the hypertonic glucose solution generated osmotic pressure differences (ΔP) averaging 1.70 ± 0.07, 1.69 ± 0.08 and 1.34 ± 0.05 cm H_2_O (N = 8). These results are shown in Figure 2. The ANOVA analysis showed that a significant difference existed in these three groups (P = 0.002). Subsequently, student t-tests were carried out to compare the three pairs, PLPC and DPPC, POPC and DPPC, and PLPC and POPC, separately. There were significant differences between POPC and DPPC (p = 0.002), and PLPC and POPC (p = 0.003). There was no significant difference between PLPC and DPPC (p = 0.80).

## Discussion

Several published studies have indicated that 1) there is endogenous SAPL lining on the surface of cartilage; 2) this SAPL is a good agent for antistick and lubrication; 3) the exogenous SAPL can be reversibly adsorbed onto the surface of cartilage and 4) the adsorbed exogenous SAPL could be effective in the treatment of osteoarthritis. As mentioned earlier, recent studies have also discovered that USPC was the dominant species at two non-lung sites ie the peritoneal cavity and pleural cavity.

Our results from this study strongly indicated that the dominant PC species on the surface of cartilage were USPCs, ie DLPC, PLPC, POPC and SLPC, representing a very different SAPL profile from that of inside the lung where DPPC (SPC) is the dominant species. The most interesting and important finding from our current study was that SAPL on the surface of articular cartilage contained on average only 8% DPPC. We therefore speculate that the role of DPPC at this non-lung site might be negligible especially when compared to the fact that DPPC constitutes ~60 % of all PC species in the lung regions of the body. The present results offer further support to the opinion that USPC could be the dominant PC species in most, if not all non-lung sites.

Our findings can also be supported by the results obtained by Sarma et al. [24], in which the fatty acid concentrations were measured after separating them from their phosphatidylcholine backbones. It was found that the total percentage of all *saturated *fatty acids was about 39% and the majority of fatty acids were *unsaturated *fatty acids (61%). As two fatty acids are needed to form an intact SPC or USPC molecule, DPPC requires two *saturated *fatty acids, ie palmitic acid. PLPC, POPC and SLPC require one *saturated *fatty acid, either palmitic or stearic acid, and one *unsaturated *fatty acid, either linoleic or oleic acid. In the case of DLPC it requires two *unsaturated *fatty acids, ie linoleic acid. By following this rule, the percentages of total *saturated *and *unsaturated *fatty acids in our PC samples can be calculated to be around 42% and 58% respectively, which were very close to those reported in the study mentioned above [24].

On the basis of this species identification, we proceeded to carry out semipermeability studies on two USPC species, ie PLPC and POPC, and compared them to DPPC. Statistically, no difference was found in mean osmotic pressure differences (ΔP) between PLPC and DPPC (p = 0.80), though there was a significant difference between POPC and DPPC (p = 0.002). However, the mean osmotic pressure differences (ΔP) among these three SAPL were very similar. These results were encouraging, in that they demonstrate that PLPC and POPC, the two dominating USPC species on the surface of cartilage, have equivalent or similar semipermeability properties to that of DPPC. This finding plus our previous findings [21-23] are important because we now know that the endogenous SAPL species inside the joint are mainly USPC and these USPC have properties of antistick, lubrication and semipermeability.

DPPC is the main PC species in lung SAPL or surfactant. The obvious reason for this is that DPPC has a gel-liquid crystal transition temperature of 41.5°C, which effectively makes it a rigid molecule at body temperature and is therefore more capable in reducing surface tension. At non-lung sites, surface tension reduction is not a physiological requirement; therefore, it is not surprising to find out that the relative quantity of DPPC in total PC species found at non-lung sites such as in the eustachian tube, stomach, peritoneal cavity and pleural cavity are all much lower than that of the lung [19-23,28].

Results from our current and previous studies indicate that SAPL, especially the USPC species i.e. PLPC and POPC, could be the important components in maintaining normal physiological functions of joint cartilage. The SAPL molecule is actually a zwitterion containing a strongly positively charged quaternary ammonium ion at one end which could enable it to bind to most epithelial surfaces which are negatively charged [29]. Besides the confirmed anti-friction/lubrication properties [21], these USPC species could also be an important component of the whole semipermeability system in regulating water transport in the joint by strongly binding to negatively charged proteoglycans. In addition, the SAPL lining that covers the intracellular gaps may be a necessity for the whole semipermeability system to be functional because proteoglycans alone may not be sufficient.

Based on the research data we have obtained so far we believe that it is worthwhile to carry out animal studies to further test the efficacy of USPC-containing SAPL samples for their properties of lubrication and semipermeability.

## Competing interests

The author(s) declare that they have no competing interests.

## Authors' contributions

YC contributes to the conception and design, the conduction of the experiment, the collection, analysis and interpretation of data, the drafting the manuscript and acquisition of funding.

RWC contributes to the analysis and interpretation of data, drafting the manuscript and acquisition of funding.

AO contributes to the analysis and interpretation of data, drafting the manuscript and acquisition of funding.

All authors have read and approved the final manuscript.
